# Level of Palliative Care Complexity in Advanced Cancer Patients: A Multinomial Logistic Analysis

**DOI:** 10.3390/jcm9061960

**Published:** 2020-06-23

**Authors:** Maria Isabel Carrasco-Zafra, Rafael Gómez-García, Ricardo Ocaña-Riola, Maria Luisa Martín-Roselló, Encarnación Blanco-Reina

**Affiliations:** 1 Fundación Cudeca, 29631 Málaga, Spain; maribelcarrasco@cudeca.org (M.I.C.-Z.); rafaelgomez@cudeca.org (R.G.-G.); marisamartin@cudeca.org (M.L.M.-R.); 2 Instituto de Investigación Biomédica de Málaga-IBIMA, 29010 Málaga, Spain; 3 Escuela Andaluza de Salud Pública, 18011 Granada, Spain; ricardo.ocana.easp@juntadeandalucia.es; 4 Instituto de Investigación Biosanitaria ibs.GRANADA, 18012 Granada, Spain; 5 International Collaborative for Best Care for the Dying Person, Liverpool L3 9TA, UK; 6 Pharmacology and Therapeutics Department, School of Medicine, University of Málaga, 29016 Málaga, Spain

**Keywords:** palliative care, complexity, advanced cancer, health care systems

## Abstract

The current treatment approach for patients in palliative care (PC) requires a health model based on shared and individualised care, according to the degree of complexity encountered. The aims of this study were to describe the levels of complexity that may be present, to determine their most prevalent elements and to identify factors that may be related to palliative complexity in advanced-stage cancer patients. An observational retrospective study was performed of patients attended to at the Cudeca Hospice. Socio-demographic and clinical data were compiled, together with information on the patients’ functional and performance status (according to the Palliative Performance Scale (PPS)). The level of complexity was determined by the Diagnostic Instrument of Complexity in Palliative Care (IDC-Pal©) and classified as highly complex, complex or non-complex. The impact of the independent variables on PC complexity was assessed by multinomial logistic regression analysis. Of the 501 patients studied, 44.8% presented a situation classed as highly complex and another 44% were considered complex. The highly complex items most frequently observed were the absence or insufficiency of family support and/or caregivers (24.3%) and the presence of difficult-to-control symptoms (17.3%). The complex item most frequently observed was an abrupt change in the level of functional autonomy (47.6%). The main factor related to the presence of high vs. non-complexity was that of performance status (odds ratio (OR) = 10.68, 95% confidence interval (CI) = 2.81–40.52, for PPS values < 40%). However, age was inversely related to high complexity. This study confirms the high level of complexity present in patients referred to a PC centre. Determining the factors related to this complexity could help physicians identify situations calling for timely referral for specialised PC, such as a low PPS score.

## 1. Introduction

The aim of palliative care (PC) is to improve the quality of life of patients and their families faced with advanced-stage life-threatening diseases [1]. Such interventions have proven effective in this regard [2,3] and, moreover, reduce the symptom burden [2,4]. PC is associated with improved care planning [2], greater patient and caregiver satisfaction with the care provided and lower levels of healthcare uptake [2,5,6].

The progressive aging of the population has increased the number of patients with chronic and advanced-stage diseases. In high-income countries, 69–82% of the population need PC before death, with dementia and cancer accounting for most of the growth in this need [7,8]. However, current resources do not allow all patients with palliative needs to be adequately cared for by specialised PC teams, and in many cases healthcare providers are overwhelmed by the demand [8,9,10]. The inability to provide the end-of-life healthcare required is a major challenge to healthcare systems. Adding to the difficulty of the situation, complex clinical situations may arise during the course of the disease, requiring specialised resources. Early, accurate identification of such situations is essential [11,12,13,14]. In view of these considerations, it has been observed that the healthcare needs of patients and their families should be addressed by a model based on shared care. In other words, different levels and types of healthcare should be integrated and coordinated [11,12]. In such a system, initial PC would be provided by primary care services (family physician and home nursing) with basic palliative-medical qualifications, while more advanced needs would be met by PC specialists. However, in clinical practice, it is often difficult to identify patients who would profit from the provision of advanced resources, and the decision is often taken subjectively, according to the competence and beliefs of the medical professional attending the patient.

From an organisational standpoint, current trends in PC include the development of complementary models of attention based on levels of complexity. Although there is no standard definition of complexity, it seems clear that it is a multifactorial concept which not only encompasses patient-dependent elements, but is influenced by other aspects of the environment [14,15,16]. The Spanish National Health System has defined complexity as the set of factors presenting greater difficulty or intensity of needs, usually requiring the intervention of specialised PC services. It depends on the characteristics of the patient, on specific (sometimes difficult) problems, on the need for diagnostic or therapeutic action and on the family’s ability to adapt to the patient’s situation [17]. In order to provide appropriate individualised care and to coordinate the suitable health resources, it is essential to use standardised tools to establish the level of complexity presented [10,16,18].

In recent years, various studies have been undertaken to address the issue of complexity in PC. Some have taken a more theoretical approach, proposing models to clarify the issues involved [14,15,16,19], while others have proposed operative criteria for assessing complexity and the coordination of resources [20,21]. In this respect, and as part of the Andalusian Palliative Care Plan, the Diagnostic Instrument for Complexity in Palliative Care (IDC-Pal©) [22] has been developed and is in use in various healthcare environments [23,24,25]. This diagnostic tool evaluates a series of complexity elements, grouped into three dimensions (patient, family-social environment and healthcare organisation), on the basis of which the level of complexity is categorised as highly complex, complex or non-complex. This classification facilitates the decision-making task; for example, a highly complex situation would require the intervention of advanced or specific PC resources, while these would not be necessary in non-complex situations. Access to advanced resources should be based on the level of complexity, and appropriate assessment and referral protocols should be established. Knowing how to identify highly complex situations will allow for the early provision of those treatments and/or interventions by the interdisciplinary team that are more precise, in order to avoid unnecessary suffering for the patient and his/her family. The aim of this study is to describe the level of complexity presented by patients attended to at a PC centre, to determine which elements of complexity are most prevalent and to identify the factors that may be associated with PC complexity in advanced-stage cancer patients.

## 2. Patients and Methods

### 2.1. Study Design, Setting and Participants

This observational retrospective study was carried out at the Cudeca Hospice in Málaga (Spain), which is integrated into the public health system. On average, it provides PC to 1100 patients each year, via interdisciplinary teams composed of doctors, nurses, psychologists and social workers. The health resources at this hospice are an inpatient unit with nine rooms, six home PC teams and a day-care unit. The following inclusion criteria were applied: persons aged 18 years or older, with advanced-stage cancer and who died between 1 August 2017 and 31 March 2018. All had been attended by the Cudeca Hospice, either at home or as inpatients.

### 2.2. Data Collection and Measures

The medical records of all the patients included in the study were reviewed, and the following data, obtained from the first clinical evaluation carried out by the PC team, were recorded for analysis: sociodemographic variables (age, sex and marital status); oncological variables (primary origin of the cancer, its extension and treatment received); functional status (according to the Barthel Index); performance status (according to the palliative performance scale (PPS)); and symptoms observed (pain, asthenia, anorexia, nausea-vomiting, constipation, dyspnoea, somnolence, insomnia, anxiety, depression). Information was also recorded on the service responsible for referring the patient to the PC programme, the characteristics of the main caregiver (sex, relationship) and the patient’s place of death.

The Barthel Index is an ordinal scale used to measure performance in activities of daily living (ADL), according to the degree of assistance required to perform ten actions related to mobility and self-care. On this scale, the higher the score, the greater the ability to function independently, as follows: total dependence (<20), highly dependent (20–39), partially dependent (40–59), minimally dependent (60–79) and independent (80–100) [26].

The PPS, a modification of the Karnofsky performance scale, is a useful tool for measuring the progressive physical decline of PC patients. It has five functional dimensions: ambulation, activity level and evidence of disease, self-care, oral intake and level of consciousness. The PPS has eleven levels, ranging from 100% (fully ambulatory and healthy) to 0% (death) in 10% steps. Each step represents a significant decrease in physical function [27]. 

The IDC-Pal© was used to measure the level of complexity presented, after the preliminary multidimensional assessment of the patient and his/her caregiver(s). The IDC-Pal© consists of 35 elements, 15 of which reflect a situation that is highly complex, while 20 represent one that is complex (Table S1). The instructions for the correct use of the instrument include a glossary to ensure that the elements identified accurately represent the contents referred to. When one or more high complexity items (HCI) are observed, the PC situation is classed as highly complex; when there are no HCIs, but at least one complex item (CI), the situation is classed as complex; and when neither HCI nor CI are observed, the situation is classed as non-complex [22].

### 2.3. Statistical Analysis

A descriptive analysis was performed to establish the main sociodemographic and clinical characteristics presented by each patient. The baseline data are summarised by numerical data (mean, standard deviation and range) for the quantitative variables and by frequency tables for the qualitative variables. 

A multinomial logistic regression model was used to study the relationship between the independent variables and the level of complexity established (i.e., situations classed as complex or highly complex, taking non-complexity as the reference standard) [28]. The independent variable PPS was classified by degree of functional deterioration, as follows: ≤40%, 50–60% and ≥70%. A 5% significance level was assumed to indicate statistical significance. Statistical data analysis was performed using SPSS version 23.0 (IBM SPSS Statistics, Armonk, NY, USA).

### 2.4. Ethical Considerations

The present study was approved by the Málaga Clinical Research Ethics Committee (project code EBR-SED-2017-1). The provisions of the Declaration of Helsinki, revised in 2013, regarding ethical principles for research on human beings, were fully complied with throughout this study. 

## 3. Results

### 3.1. Characteristics of the Study Population

The study population was composed of 501 patients, with a mean age of 71 years (standard deviation 12.3, range 33–97), of whom 60.5% were male. Most were married or had a partner (61%). Of the main caregivers, 72% were women, although 6.1% of patients had no caregiver. The most frequent patient-caregiver relationship was that of a couple (37.2%) or parent-son/daughter (34.3%). Most of the patients had been referred for PC by the oncology service (66.5%), followed by other medical specialities (such as internal and digestive medicine) (18.9%) and primary care centres (14.6%). The main characteristics of the study population are detailed in Table 1.

The most prevalent primary tumours were gastrointestinal (33.8%) and pulmonary (23.8%). Metastatic dissemination was present in 76.8% of the patients. One third had not received any specific treatment for their cancer.

The Barthel Index score presented by 53.7% of the patients indicated minimal or no dependence for ADL, but 20%, at the first assessment, were found to be totally dependent. According to the PPS, almost half of the patients presented intermediate scores, while 26.8% obtained scores <40%. The most prevalent symptoms were pain (52.6%), asthenia (35.6%) and constipation (28.3%). Over half of the patients died during hospitalisation, either in a public hospital (29.6%) or in the admission unit at the Cudeca Hospice (23.7%) (Table 1).

### 3.2. Assessment of Complexity Level in Palliative Care

According to the IDC-Pal©, the situation of most of the patients considered was classed as complex or highly complex (44.0% and 44.8%, respectively). Only 11.2% presented none of the criteria for complexity. Each patient presented 0–8 CIs or HCIs, with an average of 1.8 (SD 1.3) and a median value of 2. Five or more elements of complexity or high complexity were observed in 22 patients.

The HCIs most frequently recorded were the lack or insufficiency of family support and/or caregivers (24.3%), the presence of difficult-to-control symptoms (17.3%) and the appearance of problematic clinical situations due to cancer progression (11.2%) (Table 2). The most prevalent CI was an abrupt change in the level of functional autonomy (47.6%), followed by social-family roles being performed by the patient (15.6%), severe constitutional syndrome (13.1%) and patient-family communication conflicts (11.8%) (Table 3). Some potential CIs did not affect the patients included in the present study, such as the application of difficult-to-manage palliative sedation or the existence of limited professional competence to address problematic situations.

Among the study population, the patients in situations of high complexity, according to IDC-Pal©, were more likely to die in hospital than those in non-complex situations (64.3% vs. 51.9%, respectively).

### 3.3. Factors Related to Complexity Level

To further examine the impact of the independent variables on the PC complexity categories, a multinomial logistic regression analysis was performed. This analysis showed that the main factor related to high vs. non-complexity is the performance status. Thus, a patient whose PPS is <40% is ten times more likely to present a situation of high complexity (odds ratio (OR) = 10.68, 95% confidence interval (CI) = 2.81–40.52), while a PPS of 50–60% triples this risk (OR = 3.27, 95% CI = 1.45–7.35) (reference level: PPS >70%). However, age is inversely related to high complexity. All other variables being equal, for each additional year of life, the odds of the patient having a highly complex palliative situation decrease by 4% (Table 4).

The presence of complexity with respect to non-complexity is also related to the PPS; patients who start PC with PPS <40% are eight times more likely to have a complex situation (OR = 7.98, 95% CI = 2.11–30.22). In addition, the presence of dyspnoea in the initial PC assessment triples the odds of complexity (OR = 3.24, 95% CI = 1.06–9.91). A similar pattern for the age effect was observed, but in this case the difference was not statistically significant (Table 4). Neither the patient’s sex, nor the presence of metastases, that of other frequent symptoms (pain and asthenia), the level of health care provided or the type of medical service referring the patient to the PC centre were predictors of complexity.

## 4. Discussion

The main aim of this study is to determine the levels of complexity presented by advanced-stage cancer patients attended to at a PC centre and to identify factors that may be associated with this complexity. According to the IDC-Pal© results obtained, 88.8% of the study population presented at least one CI or HCI. Among the complexity criteria most frequently observed were those corresponding to the clinical dimension; many patients underwent an abrupt decrease in their level of functional autonomy (CI) and presented difficult-to-control symptoms (HCI). These results are consistent with those of previous research, in which the presence of physical symptoms is the most frequently-cited criterion for the use of specific PC resources [21,24,29]. However, it is interesting to note that up to a third of the patients in our study population presented one or more elements of complexity related to the family and to the patient’s environment; in this respect, the absence or insufficiency of family support and/or caregivers was most commonly observed.

To date, very few studies have been undertaken to assess the level of complexity in PC. However, within this limited research, two teams of researchers, also using IDC-Pal©, have detected high complexity in around 65% of the patients considered. In both cases, the items most frequently identified coincide with those obtained in the present study (i.e., an abrupt fall in the level of functional autonomy, the appearance of difficult-to-control symptoms and the social-family role performed by the patient) [23,24]. Another study proposed a predictive model of complexity in PC (PALCOM) to identify multidimensional variables that influence the level of complexity in patients with advanced-stage cancer. According to these authors, 41% of their patients presented high levels of PC complexity and in another 42.9% the complexity was classed as “medium” [21]. In view of these findings, and our own study results, we conclude that there is a significant prevalence of moderate and high levels of complexity among these types of patients, and that in most cases their referral to the PC centre is appropriate and timely.

The factors most strongly related to the level of complexity are performance status, age and the presence of dyspnoea. According to our multinomial logistic regression model, the most consistent predictor of complex and highly complex situations is the performance status; thus, PPS values <40% increase the likelihood of moderate or high levels of complexity by 8 and 10 times, respectively. This association is a novel finding; to our knowledge, the previous use of PPS has mainly been limited to the initial assessment for an end-of-life prognosis and for the prediction of survival [30,31]. However, we believe this finding is logical and consistent with the fact that the sudden appearance of disabling functional impairment is the most frequent element of complexity among the patients studied. Regular, systematic assessment by the PPS could facilitate the prompt identification of any decline in performance status and enable timely referral for supportive and PC services [32], thus enhancing patients’ quality of life [2,3,33]. In practical terms, as soon as a patient’s PPS score falls below about 40–50%, this would suggest the time is appropriate to review options for end-of-life care to be provided by specialised PC teams.

Among the physical symptoms most frequently observed at the initial assessment of the patients in our study (pain, dyspnoea and asthenia), the most significant is dyspnoea, the presence of which triples the likelihood of complexity. The complex biopsychosocial aetiology of this symptom and its manifestations make it particularly challenging to control [34]. Dyspnoea is very distressing for patients and their families, and in itself may have a secondary impact on functional status.

Our investigation revealed an inverse relationship between advanced age and the likelihood of high complexity. This finding corroborates the previous work of Hodiamont, who reported that the patient’s age is a factor that may influence the complexity of a PC situation [14]. We believe the association between age and complexity may arise from the greater conformity and adaptation of older patients and their family environment to the situation of terminal illness.

The need for specialised PC and the intensity of the intervention required depend mainly on the palliative complexity presented by the patient. However, most previous studies have focused on the detection of needs, in order to identify patients who would benefit from end-of-life PC, but have not taken into consideration the degree of complexity that may be present. As regards the detection of needs, diverse criteria have been proposed [35,36,37] and measurement instruments have been devised, including the Scottish Prognostic Indicator Tool (SPICT) [38], the Gold Standards Framework Prognostic Indicator Guidance (PIG-GSF) [39] and the NECPAL (palliative needs) instrument [40]. With respect to patients and their families, complexity involves aspects such as age, background, comorbidity, the intensity of symptoms, functional, emotional and social status, prognosis, family members’ ability to care for the patient and the interaction among all these elements. Further complexity can arise according to the interventions proposed and implemented, in areas such as their intensity, duration and diagnostic or therapeutic difficulty, the resources needed and problems affecting decision making [18].

Much has been written regarding the need for PC and the benefits of earlier referral, but little research has been conducted regarding the use of screening tools to improve the identification of patients in need of more resource-intensive specialised PC, distinguishing them from those for whom a more generalist approach is sufficient [37]. The aspect of particular interest in the present study is that it analyses the results obtained for a sizeable population from the use of the IDC-Pal©, an instrument that has been developed to evaluate PC complexity comprehensively, rapidly and objectively. We believe the use of this instrument could improve the quality of PC, facilitating the use of a shared-care model applied according to the degree of complexity presented. Moreover, the use of IDC-Pal© throughout the follow-up process would enable managers to detect and react to the changing complexity of the case (taking into account that the severity of the case and the patient’s needs may vary during the course of the illness).

The present study has certain limitations that should be acknowledged. The study population was selected from a single healthcare environment, a circumstance that might restrict its external validity. Nevertheless, we believe the patients examined in this study are representative of the population of advanced-stage cancer patients in a specialised PC setting. The sociodemographic data obtained, the prevalence of primary cancer and the presence of symptoms are all comparable with previously-reported data [41,42,43]. Another limitation to this study is its cross-sectional design, which does not allow causal relationships to be established, although it can detect factors related to complexity. In addition, the data collection were retrospective, which made them dependent on the quality of the information present in the medical records consulted. Furthermore, although the type and magnitude of complexity may change in the course of the disease, it was only evaluated in the first assessment.

On the other hand, our research also has significant strengths, especially the fact that the study population was considerably larger than those analysed in previous studies of complexity [21,23,24,41,42,43]. In consequence, a substantial and diverse body of clinical and functional data was collected, underpinning the statistical analysis performed. In our opinion, this type of study helps identify items and clinical conditions that may be predictive of complexity and, therefore, can help decision-making regarding the optimum time for referring the patient for specialised PC, as well as assisting intra-case management.

## 5. Conclusions

In conclusion, this study confirms the high level of complexity presented by many patients referred to a PC centre. Although the elements of complexity found to be most prevalent are those related to the patients’ clinical evolution, aspects of their socio-family environment also play a significant role. Greater knowledge of factors related to this complexity could help identify signals that would enable clinicians to improve the timing of referral for specialised PC. In our opinion, clinicians should take into account the complexity of the situation when PPS is less than 40%. Awareness of this consideration could be very useful for health professionals providing end-of-life care. Finally, the use of structured instruments to identify the level of complexity presented by a patient could help clinicians decide the most appropriate intensity of intervention required within a shared-care model.

## Figures and Tables

**Table 1:** Characteristics of the study population (*N* = 501).

Variables	Value
Age, mean (standard deviation)	71.5 (±12.3)
Sex, *n* (%)	
Male	303 (60.5)
Female	198 (39.5)
Marital status, *n* (%)	
Married or with partner	292 (61.0)
Widowed	99 (20.7)
Divorced/separated	49 (10.2)
Single	39 (8.1)
Main carer, *n* (%)	
Female	323 (72.9)
Male	93 (21.0)
No carer	27 (6.1)
Relationship with carer, *n* (%)	
Partner	167 (37.2)
Child (son/daughter)	154 (34.3)
Agency referring for PC, *n* (%)	
Oncology service	274 (66.5)
Other specialities	78 (18.9)
Primary care	60 (14.6)
Primary cancer, *n* (%)	
Gastrointestinal	153 (33.8)
Lung	108 (23.8)
Breast-Gynaecological	59 (13.0)
Genito-Urinary	51 (11.3)
Head and neck	24 (5.3)
Haematological	21 (4.6)
Other	37 (8.2)
Cancer dissemination, *n* (%)	
Metastasis	365 (76.8)
Local	110 (23.2)
Cancer treatment, *n* (%)	
Yes	322 (66.4)
No	163 (33.6)
Barthel Index, *n* (%)	
100	62 (13.3)
≥60	188 (40.4)
40–55	115 (24.7)
20–35	55 (11.8)
≤20	45 (9.7)
PPS, *n* (%)	
≥70%	125 (26.0)
50–60%	227 (47.2)
≤40%	129 (26.8)
Most frequent symptoms, *n* (%)	
Pain	259 (52.6)
Asthenia	175 (35.6)
Constipation	139 (28.3)
Dyspnoea	99 (20.1)
Insomnia	92 (18.7)
Anorexia	69 (14.0)
Nausea/Vomiting	66 (13.4)
Somnolence	40 (8.1)
Depression	36 (7.3)
Anxiety	35 (7.1)
Place of death, *n* (%)	
Home	234 (46.7)
Public hospital	148 (29.6)
Inpatient Unit at Cudeca	119 (23.7)

PPS: Palliative Performance Scale.

**Table 2:** Number of patients with a highly complex situation, according to the Diagnostic Instrument of Complexity in Palliative Care (IDC-Pal©).

Items of High Complexity	*N* (%)
The patient is a child or adolescent	0 (0.0)
Symptoms difficult to control	82 (17.3)
Refractory symptoms	8 (1.7)
Urgent situation in the terminal cancer patient	14 (3.0)
Last hours/days of life difficult to control	7 (1.5)
Clinical situation due to difficult-to-control cancer progression	53 (11.2)
Risk of patient committing suicide	4 (0.8)
Patient requests the process of death to be hastened	2 (0.4)
Patient presents existential distress and/or spiritual suffering	7 (1.5)
Absence or insufficiency of family support and/or caregivers	115 (24.3)
Family members and/or caregivers not competent to give care	12 (2.5)
Dysfunctional family	6 (1.3)
Family and/or caregiver burden	6 (1.3)
Structural limitations of environment for the patient	8 (1.7)
Application of palliative sedation difficult to manage	0 (0.0)
Total of patients with at least one item of high complexity	216 (44.8)

**Table 3:** Number of patients with a complex situation, according to IDC-Pal©.

Items of Complexity	*N* (%)
The patient is a healthcare professional	3 (0.6)
Social-family role performed by the patient	74 (15.6)
Previous physical, psychological or sensorial disability	23 (4.9)
Recent and/or active addiction problems	7 (1.5)
Previous mental illness	12 (2.5)
Acute decompensated organ insufficiency in non-oncological terminal patient	0 (0.0)
Severe cognitive failure	23 (4.9)
Abrupt change in level of functional autonomy	225 (47.6)
Presence of difficult-to-control comorbidity	19 (4.0)
Severe constitutional syndrome	62 (13.1)
Clinical management difficult due to repeated non-compliance with therapy	15 (3.2)
Communication conflicts between patient and family	56 (11.8)
Communication conflicts between patient and healthcare team	17 (3.6)
Inadequate emotional coping by patient	3 (0.6)
Complex bereavement	5 (1.1)
Difficulty in the indication and/or management of medication	2 (0.4)
Difficulty in the indication and/or management of interventions	1 (0.2)
Limitations of professional competence to address situations	0 (0.0)
Difficulty in managing or acquiring instrumental techniques and/or specific material at home	3 (0.6)
Difficulty in managing coordination and logistic needs	2 (0.4)
Total of patients with at least one item of complexity	212 (44.0)

**Table 4:** Factors related to level of palliative complexity. Multinomial logistic regression for high complexity and complexity (with respect to non-complexity).

Independent Variable	OR (95% CI) High Complexity	OR (95% CI) Complexity
**Age**	0.96 (0.93–0.99) *	0.98 (0.95–1.01)
**Sex**		
Female	1.12 (0.55–2.50)	1.23 (0.56–2.70)
Male	1	1
**Agency referring for PC**		
Primary care	2.17 (0.48–9.87)	1.11 (0.24–5.10)
Oncology	1.35 (0.51–3.57)	1.26 (0.49–3.22)
Other hospital service	1	1
**Metastasis**		
Yes	1.01 (0.44–2.71)	1.27 (0.52–3.12)
No	1	1
**PPS**		
PPS < 40%	10.68 (2.81–40.52) ***	7.98 (2.11–30.22) **
PPS 50–60%	3.27 (1.45–7.35) **	3.58 (1.26–7.90) **
PPS > 70%	1	1
**Pain**		
Yes	1.26 (0.59–2.71)	0.93 (0.44–1.97)
No	1	1
**Dyspnoea**		
Yes	2.55 (0.82–7.97)	3.24 (1.06–9.91) *
No	1	1
**Asthenia**		
Yes	1.14 (0.51–2.53)	1.18 (5.54–2.61)
No	1	1

* *p* < 0.05; ** *p* < 0.01; *** *p* < 0.001. OR, Odds Ratio; 95% CI, 95% Confidence Interval; PPS, Palliative Performance Scale.

## Supplementary Materials

The following are available online at https://www.mdpi.com/2077-0383/9/6/1960/s1, Table S1: Diagnostic Tool for Complexity Classification in Palliative Care (IDC-Pal©).
