# Supplementary material for: Level of Palliative Care Complexity in Advanced Cancer Patients: A Multinomial Logistic Analysis
Source: J Clin Med. 2020 Jun 23;9(6):1960. doi: 10.3390/jcm9061960 (PMC7356562; doi:10.3390/jcm9061960)
Supplement: Supplementary file 1 [file jcm-09-01960-s001.pdf]

Supplementary materials

Table S1. Diagnostic Tool for Complexity Classification in Palliative Care (IDC-Pal©).

		Items	Complexity level*	Yes	No
1. Patient	1.1 History	1.1a The patient is a child or adolescent	HC		
		1.1b The patient is a healthcare professional	C		
		1.1c Social-family role performed by patient	C		
		1.1d Previous physical, psychological or sensorial disability	C		
		1.1e Recent and/or active addiction problems	C		
		1.1f Previous mental illness	C		
	1.2 Clinical Situation	1.2a Symptoms difficult to control	HC		
		1.2b Refractory symptoms	HC		
		1.2c Urgent situations in the terminal cancer patient	HC		
		1.2d Last hours/days of life difficult to control	HC		
		1.2e Clinical situations due to cancer progression difficult to control	HC		
		1.2f Acute decompensated organ insufficiency in non-oncological terminal patient	C		
		1.2g Severe cognitive failure	C		
		1.2h Abrupt change in level of functional autonomy	C		
		1.2i Presence of comorbidity difficult to control	C		
		1.2j Severe constitutional syndrome	C		
		1.2k Clinical management difficult due to repeated non-compliance with therapy	C		
	1.3 Psycho-Emotional	1.3a Risk of patient committing suicide	HC		
		1.3b Patient is asking to hasten the process of death	HC		
		1.3c Patient presents existential anguish and/or spiritual suffering	HC		
		1.3d Communication conflicts between patient and family	C		
		1.3e Communication conflicts between patient and healthcare team	C		
		1.3f Inadequate emotional coping by patient	C		
2.Family and enviroment		2.a Absent or insufficient family support and/or caregivers	HC		
		2.b Family members and/or caregivers not competent to give care	HC		
		2.c Dysfunctional family	HC		
		2.d Family and/or caregiver burden	HC		
		2.e Complex bereavement	C		
		2.f Structural limitations of environment for the patient	HC		
3. Healthcare Organization	3.1 Professionals /Team	3.1a Application of palliative sedation difficult to manage	HC		
		3.1b Difficulty in the indication and/or management of medication	C		
		3.1c Difficulty in the indication and/or management of interventions	C		
		3.1d Limitations of professional competence to address situations	C		
	3.2 Resources	3.2a Difficulty managing or acquiring instrumental techniques and/or specific material at home	C		
		3.2b Difficulty managing coordination and logistic needs	C		

*Score	Items present	Specialized palliative care team
Highly complex	At least one high-complex item (HC)	Highly recommended

Complex	At least one complex item (C)	It might constitute a benefit
Non-complex	None of the items	Not recommended
